# *Cyclocarya paliurus* leaves alleviate high-sucrose diet-induced obesity by improving intestinal metabolic disorders

**DOI:** 10.18632/aging.205657

**Published:** 2024-03-14

**Authors:** Ye Yao, Xiaojuan Wang, Dongyu Li, Shujuan Chen, Chengjie Li, Haiyu Guan, Dongsheng Wang, Xiaoli Nie

**Affiliations:** 1 Department of Nephrology, Integrated Hospital of Traditional Chinese Medicine, Southern Medical University, Guangzhou 510315, China; 2 Institute of Integrated Traditional Chinese and Western Medicine, Xiangya Hospital, Central South University, Changsha 410008, China

**Keywords:** high-sucrose diet, obesity, gut microbiota, intestinal metabolites, *Cyclocarya paliurus*

## Abstract

High-sucrose diets are common in daily life but harmful to human health. *Cyclocarya paliurus* leaves (CPL) are a kind of tea used to alleviate metabolic diseases and are widely used in China. However, the effects of CPL on high-sucrose-induced obesity are unknown. This study aimed to describe the changes in gut metabolism induced by a high-sucrose diet and to reveal the potential mechanisms through which CPL alleviate high-sucrose diet-induced obesity. A high-sucrose-induced obesity model was generated in C57BL/6J and KM mice. The effects of CPL on obese mice were evaluated, and changes in the gut microbiota and intestinal metabolites induced by CPL treatment were observed. Furthermore, the fecal microbiota transplantation (FMT) method was used to prove that the effects of CPL on high-sucrose induced obesity depend on the changes of gut microbiota. The results of the C57BL/6J mouse experiment revealed that high-sucrose intake induced fat deposition and altered the gut microbiota. CPL treatment decreased fat deposition and alleviated disorders of the gut microbiota. Furthermore, CPL treatment increased the utilization of amnio acids, long fatty acids and saccharides and produced more bile acids, indole derivatives and less trimethylamine (TMA). A confirmatory experiment in KM mice also revealed that CPL can alleviate obesity, ameliorate intestinal metabolic disorders, and upregulate the expression of tight junction proteins in the intestinal mucosa. These results demonstrated that CPL could prevent high sucrose-induced obesity and generate more beneficial intestinal microbial metabolites but less harmful intestinal microbial metabolites.

## INTRODUCTION

Reducing the consumption of sugars to less than 25 g/day is recommended to reduce adverse effects; however, the actual intake of sugars is markedly greater than the recommended health standards for many people [1]. Recently, increasing amounts of attention have been given to high sugar intake, which leads to a greater incidence of obesity [2–4]. The prevalence of obesity continues to increase worldwide, and more than one billion individuals worldwide are currently obese [3]. The consensus that obesity induces a series of diseases, including type 2 diabetes mellitus (T2DM), nonalcoholic fatty liver disease and cardiovascular disease, has been accepted by the public [5]. Increased consumption of sugars with a high content of energy is the main environmental factor contributing to this dramatic increase in obesity, which has reached epidemic levels [3].

Sucrose is a safe and inexpensive sweetener composed of a molecule of glucose and a molecule of fructose, and it is widely consumed to make delicious food in daily life [3]. In China, sucrose consumption has shown an overall upward trend, and the consumption of high-sucrose diets, including snacks, fast food, and beverages, has increased significantly [6]. A high-sucrose diet not only increases the intake of energy but also disorders the intestinal microbiome [6, 7]. Previous studies have shown that high-fat diets remodel the gut microbiome, which in turn causes gut metabolites to develop in ways that are harmful to human health [7]. However, studies on the influence of a high-sucrose diet on the intestinal microecological balance are rare. The constitution of the gut microbiota is regulated by diet, and nutrient metabolism is influenced by different conditions in the gut microbiota [7, 8]. Hence, we infer that a high-sucrose diet markedly changes the intestinal microenvironment.

*Cyclocarya paliurus (Batal.)* Iljinsk (*C. paliurus*), commonly called the “sweet tea tree”, is grown only in southern China [9]. Since the 1970 s, the mystery of *C. paliurus* leaves (CPL) has been unveiled, and CPL were gradually adopted by ordinary people as a health care tea with a special flavor and benefits for obese and diabetic conditions [10]. The safety and efficacy of CPL are widely recognized by the US, which was the first health tea approved by the U.S. Food and Drug Administration from China in 1999 [10]. Furthermore, CPL were approved as a new raw food material by The National Health Commission of the People’s Republic of China in 2013 [10]. Many studies have attempted to explain the mechanism by which CPL and their extracts improve glucose and lipid metabolism [11–14], but the results have been unsatisfactory.

Our previous study demonstrated that *C. paliurus* polysaccharides (CCPPs) alleviate type 2 diabetes symptoms by modulating the gut microbiota and SCFAs [15]. Although the pharmacologic actions of C. *paliurus* are controversial, improving energy metabolism by regulating the intestinal microecological balance seems to be recognized by the majority of researchers [13, 15–18]. However, studies examining the effects of CPL on high-sucrose diet-induced obesity are lacking.

In this study, we observed the effects of CPL on high-sucrose diet-induced obesity. Furthermore, changes in the gut microbiota and in the levels of all 650 different intestinal metabolites were detected after treatment with CPL. Changes in the intestinal microenvironment will be comprehensively described in this study.

## MATERIALS AND METHODS

### Chemicals and reagents

Sucrose, methanol, anhydrous ethanol, sodium hydroxide and trifluoroacetic acid were purchased from Sinopharm Chemical Reagent Co., Ltd., (Shanghai, China). The normal diet (nutritional ingredients are listed in Supplementary Table 1) was purchased from Beijing Ke-ao-xie-li Feed Co., Ltd. (China). An insulin ELISA kit (EZRMI-13 K, Merck Millipore Co., Ltd., Darmstadt, Germany) and a stool DNA kit (DP328, Tiagen Biotech Co., Ltd., Beijing, China) were used. Total cholesterol (TC, E-BC-K109-M), triglyceride (TG, E-BC-K261-M), high-density lipoprotein cholesterol (HDL-C, E-BC-K221-M), and low-density lipoprotein cholesterol (LDL-C, E-BC-K205-M) colorimetric assay kits were purchased from Elabscience Biotechnology Co., Ltd., (Wuhan, China). A reverse transcription kit was purchased from Thermo Scientific Co., Ltd., (K1622, USA), and TRIpure reagent was purchased from Aidlab Biotechnologies Co., Ltd. (Beijing, China). MonAmp™ SYBR^®^ Green qPCR mix was purchased from Monad Biotech Co., Ltd. (Zhuhai, China). Antibodies, including Claudin-1 (E-AB-12455), Occludin (E-AB-40648) and ZO-1 (E-AB-52081) were bought from Elabscience Biotechnology Co., Ltd., (Wuhan, China).

### Animal experiments

A total of 30 male SPF C57BL/6J mice (8 weeks old, 22–25 g, C57 experiment) and 24 KM mice (6 weeks, 28–32 g, KM experiment) were purchased from Hunan SJA Laboratory Animal Co., Ltd., (Changsha, China) and raised in the Department of Laboratory Animals, Central South University (Changsha, China) under a relative humidity of 50 ± 15%, a temperature of 25 ± 2°C, a 12-hour dark-light cycle with sterile water and diet. There were 5 mice living in one cage. All animal experiments were approved by the Animal Ethics Committee of Institutional Animal Care and Use Committee of Central South University (Changsha, China, permit number: 2021sydw0111).

**Figure 1:**
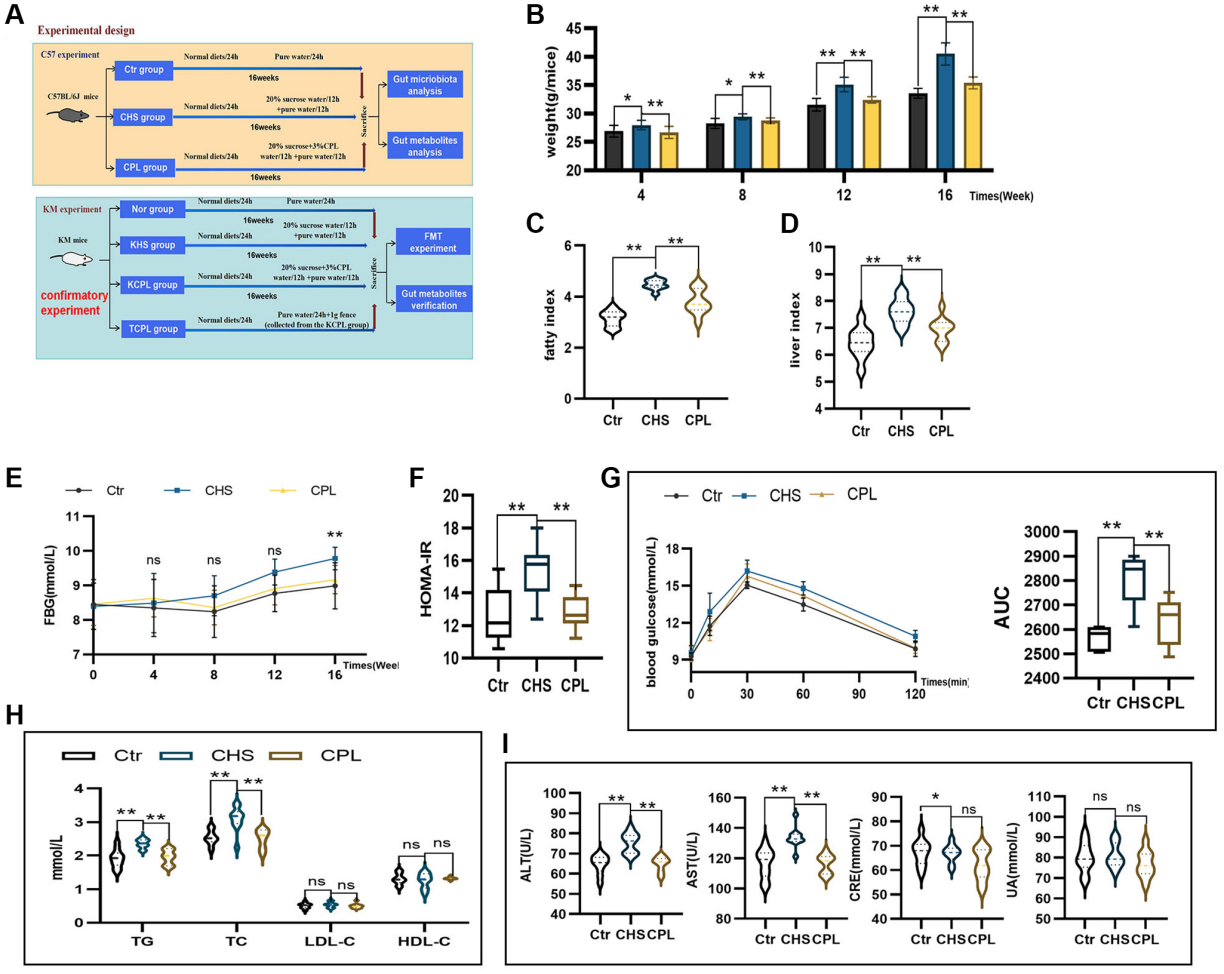
**CPL alleviate high-sucrose intake-induced fat accumulation, hyperglycemia, dyslipidemia and disorders of hepatic and renal function.** (**A**) Experimental design. (**B**) Weight of the mice. (**C**) Liver index. (**D**) Fat index. (**E**) Fasting blood glucose (FBG). (**F**) Homeostasis model assessment of insulin resistance (HOMA-IR). (**G**) The oral glucose tolerance test (OGTT) and area under the curve (AUC) were calculated for blood glucose levels during the OGTT. (**H**) Serum lipid levels. (**I**) Hepatic and renal function. *n* = 10 mice per group. The data are presented as the mean ± SEM. Statistical analysis was performed using Student’s *t* test. ^*^p < 0.05, ^**^p < 0.01, Abbreviation: ns: not significant.

Thirty C57BL/6J mice were divided into 3 groups (10 mice per group, C57 experiment): the control group (Ctr group), high-sucrose group (CHS group) and CPL group. Twenty-four KM mice were divided into 4 groups (6 mice per group; KM experiment): the normal group (Nor group), high-sucrose group (KHS group), KCPL group and TCPL group. All the mice were fed a normal diet. Mice in the Ctr and Nor groups were given normal pure water. Mice in the CHS and KHS groups were fed high-sucrose and pure water (7:00 to 19:00 pure water, 19:00 to the next day, 7:00 sucrose), and mice in the CPL and KCPL groups were given sucrose+CPL water (3 g/CPL+20 g sucrose soaked in 100 ml 100°C water, cooled at 25°C and filtered for CPL residue; 7:00 to 19:00 pure water, 19:00 to the next day, 7:00 sucrose + CPL tea). Mice in the TCPL group were given normal pure water but were treated with 1 g/kg/day feces (collected from the KCPL group). All the mice were treated for 16 weeks, after which their weight, food intake and water intake were measured every week (Figure 1A).

### Biochemical assays

Fasting blood glucose (FBG, fasting for 8 hours) levels were measured with a glucometer (Accu-Chek (R) Active, Roche Diagnostics GmbH, Mannheim, Germany) via angular vein sampling. An oral glucose tolerance test (OGTT) was performed before the mice were sacrificed, according to the methods of previous methods [19, 20]. TC, TG, HDL-C, LDL-C, and serum glucose levels were determined following the manufacturer’s instructions. Fasting serum insulin levels were quantified by ELISA kits. The homeostasis model assessment of insulin resistance index (HOMA-IR) was calculated as previously described [20].

### 16S rRNA gene sequencing analysis

Colonic content samples were collected for 16S rRNA gene analysis. Briefly, the steps of the analysis were DNA extraction, DNA quality detection, 16S rRNA gene amplification, purification of the PCR products, PE library construction, Illumina sequencing and bioinformatics analysis [20, 21]. The details are provided in Supplementary Files 1 and 3. 16S rRNA gene sequences data and the metagenomic sequences data that supported the findings of this study are publicly available at the NIH National Center for Biotechnology Information Sequence Read Archive (SRA) with PRJNA1063614.

### Quantitative analysis of intestinal metabolites

The colonic contents were collected for quantitative analysis via UHPLC-QTOF-MS technology. In the C57 experiment, 650 metabolites were detected in this study. In the KM experiment, 3 indole derivatives (including indole-3-lactic acid, indoleacetic acid and 3-indolepropionic acid), 12 bile acids (including β-MCA, MDCA, β-HDCA, CDCA, LCA, IsoLCA, CA, IsoDCA, UCA, UDCA, β-UDCA, and 3-DHCA) and TMA-related metabolites (including DTMA, TMA and TMAO) were absolutely quantified via UHPLC-QTOF-MS technology. The steps included sample preprocessing, QC preparation, standard curve preparation, LC-MS/MS mass spectrometry analysis and data processing. These steps were performed by Shanghai Applied Protein Technology Co., Ltd. The details are provided in Supplementary Files 2 and 3.

### Quantitative real-time PCR (qRT-PCR)

Total RNA was isolated from frozen colonic tissues with TRIzol reagent, and cDNA was synthesized from 1 μg of total RNA using a reverse transcription kit. qRT-PCR was performed using a CFX Connect system (Bio-Rad, USA) with MonAmp™ SYBR^®^ Green qPCR Mix [22]. The primers used for claudin-1, occludin and ZO-1 were synthesized by Sangon Biotech Technology Co., Ltd., and are listed in Supplementary Table 1.

### Western blot

The mice colon samples were lysed in RIPA buffer (Beyotime Institute of Biotechnology, Shanghai, China) containing Halt™ Protease and Phosphatase Inhibitor Single-Use Cocktail (Thermo Fisher Scientific). The protein concentrations were quantified with a BCA assay kit according to the manufacturer’s instructions (Beyotime Institute of Biotechnology). Equal protein amounts were electrophoresed on SDS-PAGE gels (Bio-Rad), transferred to a polyvinylidene fluoride (PVDF) membrane (0.22 μm, Biosharp, Anhui, China), blocked with 5% non-fat milk and incubated with Claudin-1, Occludin and ZO-1 and β-actin antibodies overnight. After incubation with the corresponding secondary antibody for 2 hours, target proteins were visualized using a ChemiDoc™ MP Imaging system (Bio-Rad) and analysed using Image Lab software (Bio-Rad) [22].

### Statistical analysis

SPSS 23.0 and R software were used for the statistical analysis. GraphPad Prism 9.0 software was used for graphical presentation. The heatmaps were drawn using R software (pheatmap package). One-way ANOVA was performed to investigate alterations among three or more groups, and Student’s *t* tests were performed to compare the data between only two groups. The data in the heatmap were normalized with the z score method (z = (*x* − μ)/σ).

## RESULTS

### CPL alleviate high-sucrose diet-induced fat deposition and metabolic disorders

Mice in the CHS and CPL groups consumed more sucrose water but consumed less food (Supplementary Figure 1A, 1B). Standard fodder, which contained 23.07% protein, 11.85% lipids and 65.08% carbohydrates, was utilized in our study (Supplementary Table 2). Hence, the intake of proteins and lipids was lower in the CHS and CPL groups than in the Ctr group (Supplementary Figure 1C, 1D). While the mice in the CHS and CPL groups consumed a large amount of sucrose water, the carbohydrate intake in those two groups was markedly greater than that in the Ctrl group (Supplementary Figure 1E). Finally, compared to that in the Ctr group, the energy intake of the CHS group increased by 39.85%, 26.83%, 33.36% and 26.78% at 4 weeks, 8 weeks, 12 weeks and 16 weeks, respectively, and the energy intake was not reduced in the CPL group (Supplementary Figure 1F).

Mice in the CHS and CPL groups consumed the same amount of food and water (dissolved sucrose), which indicated that the mice in those two groups had the same energy intake. However, compared with those in the CHS group, the weights of the mice in the CPL group gradually decreased (Figure 1B). The liver index and fat index, which reflect fat deposition, were significantly lower in the CPL group (Figure 1C, 1D). Fasting blood glucose (FBG) was tested every 4 weeks. The FBG levels of the mice in the CPL group were lower than those in the CHS group, and after 16 weeks of CPL intervention, the difference became apparent (Figure 1E). The HOMA-IR index, which reflects insulin resistance, decreased by 16.98% in the CPL group (Figure 1F). OGTT results confirmed that CPL treatment improved glucose intolerance. CPL treatment prevented a substantial increase in blood glucose levels according to the OGTT, and the AUC decreased by 6.39% (Figure 1G). TG and TC levels were also decreased after CPL treatment, but LDL-C and HDL-C levels did not markedly change (Figure 1H). The levels of transaminases, including ALT and AST, were significantly decreased after 16 weeks of CPL treatment, but the levels of CRE and UA were not significantly lower in the CPL group (Figure 1I). Taken together, these findings demonstrated that CPL could ameliorate high-sucrose-induced obesity and ameliorate metabolic disorders.

### CPL alter the composition of the gut microbiota in high-sucrose-induced obese mice

**Figure 2:**
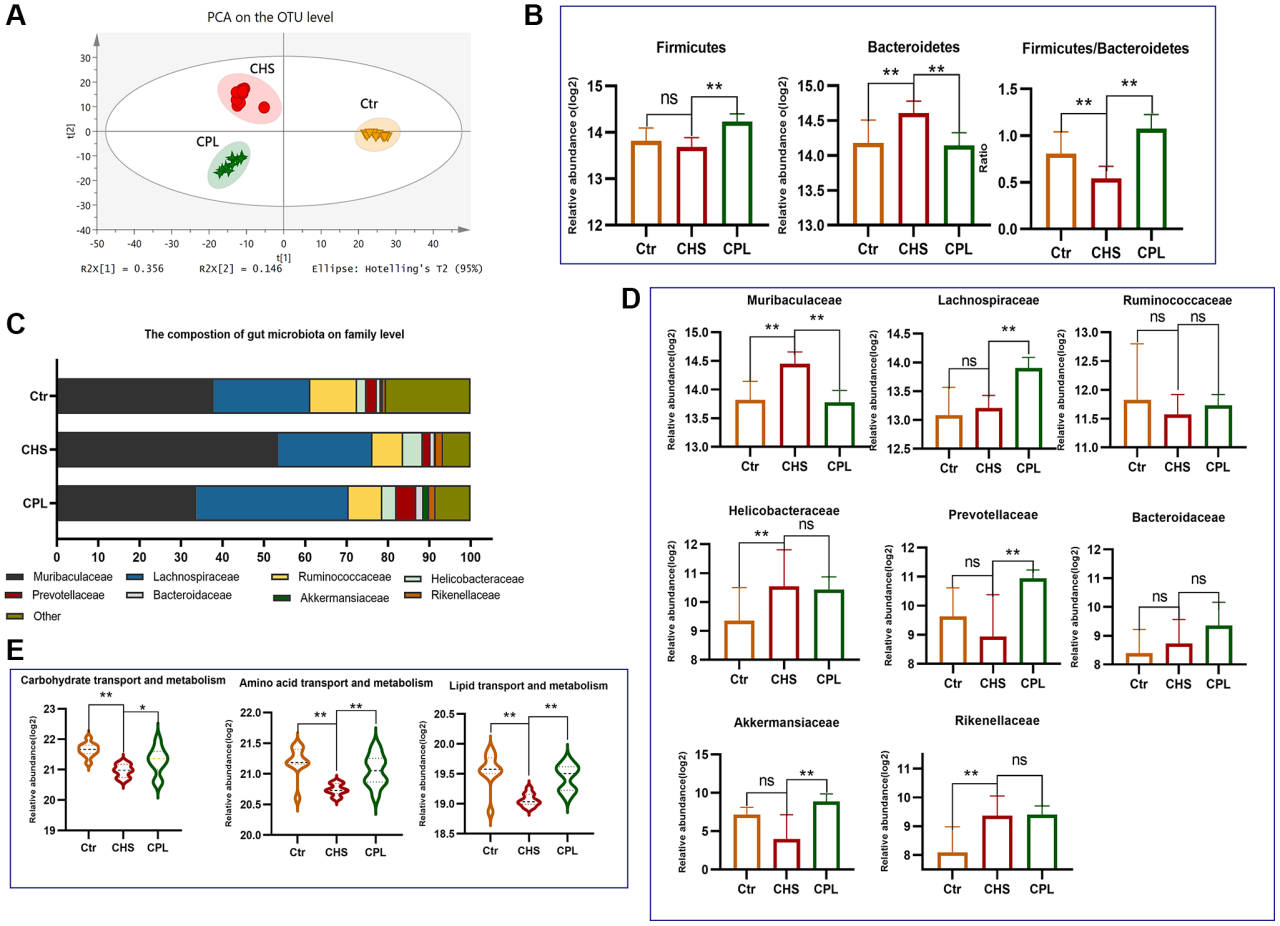
**Effects of CPL on gut microbiota modulation in high-sucrose-induced obese mice.** (**A**) Principal component analysis (PCA). (**B**) Relative abundance of the main phyla. (**C**) The composition of the gut microbiota at the family level. (**D**) Bar graph of the 8 most abundant families. (**E**) Gut microbiota functional prediction. *n* = 10 mice per group. The data are presented as the mean ± SEM. The data were log2-transformed. Statistical analysis was performed using Student’s *t* test. ^*^p < 0.05, ^**^p < 0.01, Abbreviation: ns: not significant.

After 16 weeks of CPL intervention, the gut microbiota composition of the mice in the CPL group was unique to that of the mice in the Ctr and CHS groups (Figure 2A). According to the biological classification, gut microbes can be divided into domain, kingdom, phylum, class, order, family, genus, and species. We analyzed the changes in the gut microbiota at the phylum level and found that more than 90% of the gut microbes were *Firmicutes* and *Bacteroidetes*. Long-term consumption of sucrose clearly increased the relative abundance of *Bacteroidetes* (Figure 2B). CPL treatment increased the abundance of *Firmicutes* and decreased the relative abundance of *Bacteroidetes* (Figure 2B). Correspondingly, the ratio of *Firmicutes* to *Bacteroidetes* was significantly increased by CPL treatment (Figure 2B).

At the family level, *Muribaculaceae*, *Lachnospiraceae* and *Ruminococcaceae* were the main families in all the groups (Figure 2C). Eight families accounting for more than 1% of the bacteria in each group were selected. The abundances of *Muribaculaceae* decreased in response to CPL treatment, but the abundance of *Lachnospiraceae*, *Prevotellaceae* and *Akkermansiaceae* increased in response to CPL treatment (Figure 2D). The gene expression patterns of the gut microbiota were predicted via functional COG prediction. CPL treatment increased the expression of genes related to carbohydrate transport and metabolism, amino acid transport and metabolism and lipid transport and metabolism in gut microorganisms, which indicated that CPL might increase the metabolic ability of gut microbes (Figure 2E).

### CPL affect macronutrient utilization in high-sucrose-induced obese mice

**Figure 3:**
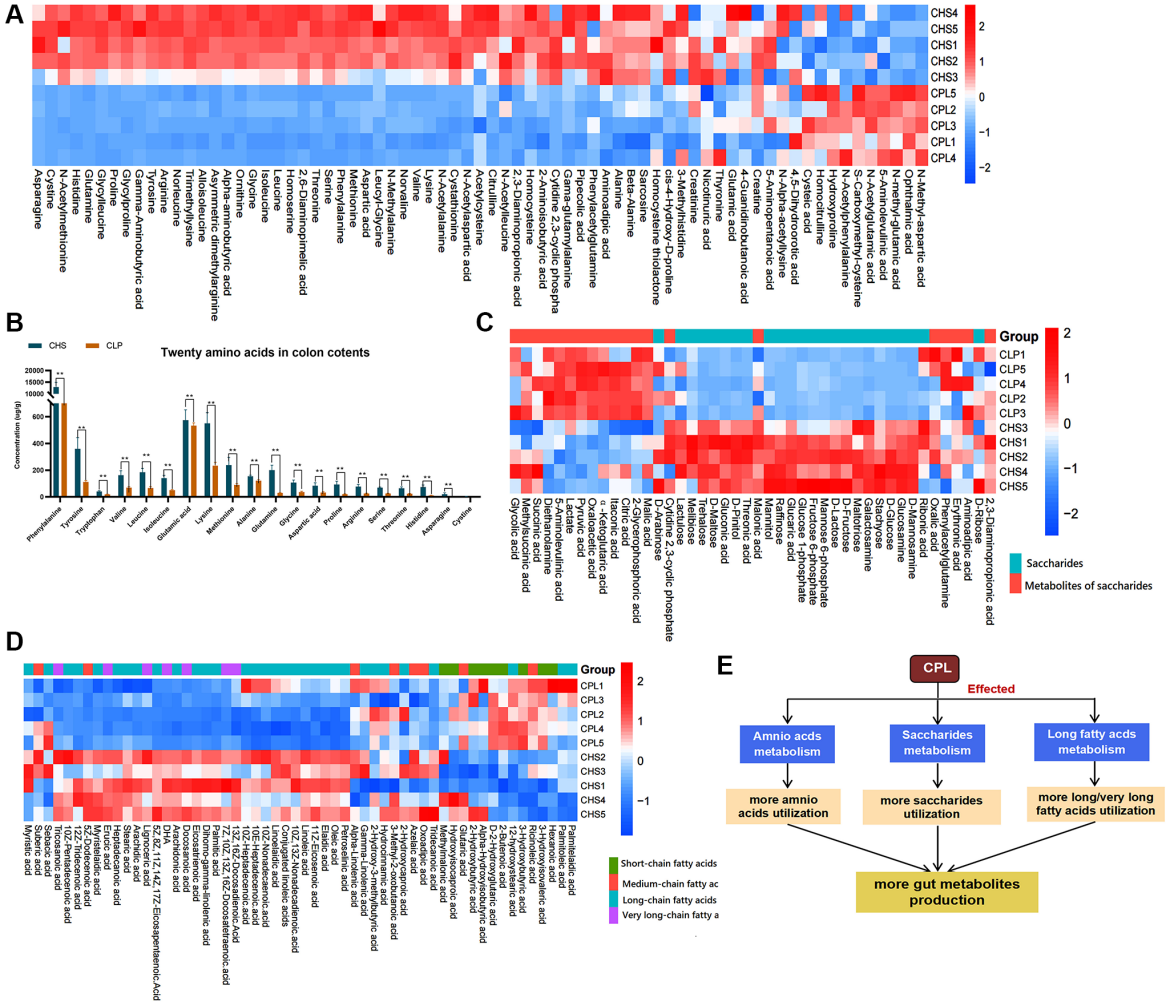
**Effects of CPL on macronutrient utilization.** (**A**) Heatmap of amino acids and derivatives. (**B**) Twenty amino acid residues in colon contents. (**C**) Heatmap of saccharides and metabolites of saccharides. (**D**) Heatmap of fatty acids. (**E**) CPL affected the utilization of the three macronutrients. The data in the heatmap were normalized by the z score method. *n* = 5 mice per group. The data in Figure 3B are presented as the mean ± SEM. Statistical analysis was performed using Student’s *t* test. ^*^p < 0.05, ^**^p < 0.01.

In the C57 experiment, we detected 650 metabolites in the colonic contents of the CHS group and the CPL group, but only 312 metabolites were absolutely quantified in this study (Supplementary File 1). Three macronutrients, amino acids and derivatives, fatty acids, and carbohydrates, were selected from the 312 different metabolites. Seventy-one amino acids and their derivatives were detected, and only 10 amino derivatives were residual more, additionally, sixty-one other amino acids and their derivatives were relatively less abundant in the CPL group (Figure 3A). Twenty amino acids that make up proteins were detected, and for all of them, there were less residual amino acids in the CPL group than that in the CHS group. The findings indicate that mice in the CPL group might utilize more amino acids (Figure 3B). Fifty-six fatty acids, including SCFAs, medium-chain fatty acids (MCFAs), long-chain fatty acids and very long fatty acids, were found. There were fewer residual long-chain fatty acids and very long fatty acids in the CPL group than in the CHS group. However, there were more residual SCFAs and MCFAs in the CPL group than in the CHS group (Figure 3D). Carbohydrates, mainly polysaccharides, are digested into short-chain saccharides by the gut microbiota. Compared to those in the CHS group, the levels of 11 short-chain saccharides in the CPL group were lower, but the levels of saccharide metabolites were higher (Figure 3C). These findings demonstrated that CPL could improve the utilization of amino acids, long/very long fatty acids and polysaccharides in mice with obesity induced by high sucrose and could result in the production of more SCFAs/MCFAs and metabolites of saccharides (Figure 3E).

### CPL affect gut tryptophan metabolism in high-sucrose-induced obese mice

**Figure 4:**
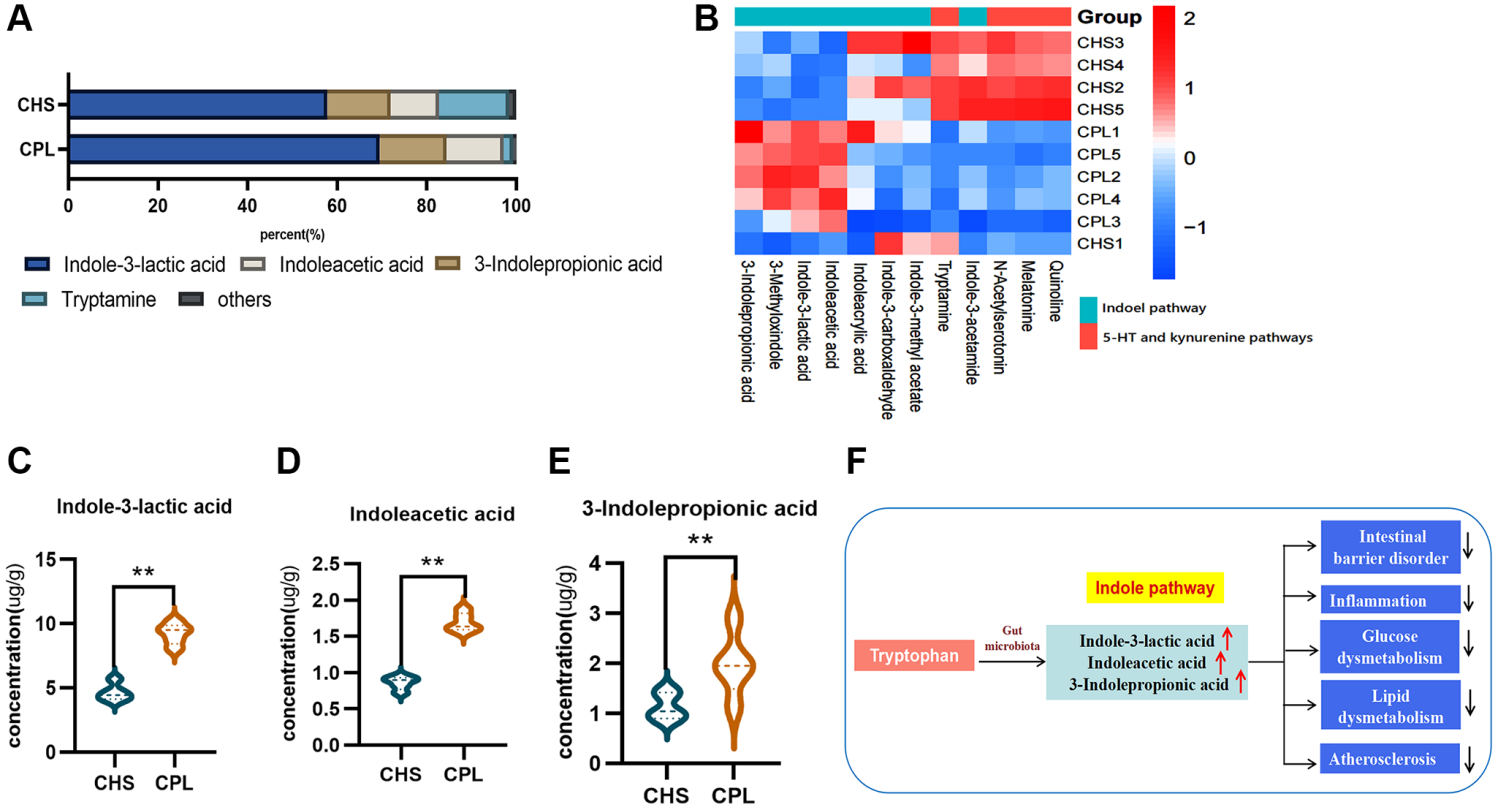
**Effects of CPL on tryptophan metabolism in the colonic contents.** (**A**) Composition of tryptophan metabolites. (**B**) Heatmap of tryptophan metabolites. (**C**) Concentration of indole-3-lactic acid. (**D**) Concentration of indoleacetic acid. (**E**) Concentration of 3-indolepropionic acid. (**F**) CPL affected indole pathway metabolism. *n* = 5 mice per group. The data are presented as the mean ± SEM. Statistical analysis was performed using Student’s *t* test. ^*^p < 0.05, ^**^p < 0.01.

Equal amounts of protein were taken from the mice in the CHS and CPL groups, but less tryptophan was detected in the colonic contents of the CPL group. These findings might indicate that CPL increase the utilization of tryptophan in the gut. Tryptophan is involved in three main metabolic pathways: the 5-HT pathway, kynurenine pathway and indole pathway. The indole pathway, activated by gut microbes, can improve intestinal barrier disorders, alleviate gut inflammation, glucose and lipid dysmetabolism, and atherosclerosis (Figure 4F) [23].

Twelve metabolites of tryptophan were detected in this study (Figure 4B). Indole-3-lactic acid, indoleacetic acid, 3-indolepropionic acid and tryptamine are the most important metabolites of tryptophan and account for more than 95% of all the metabolites in both groups (Figure 4A). CPL treatment significantly increased the concentrations of indole-3-lactic acid, indoleacetic acid and 3-indolepropionic acid (Figure 4C–4E).

### CPL affect gut bile acid metabolism in high-sucrose-induced obese mice

**Figure 5:**
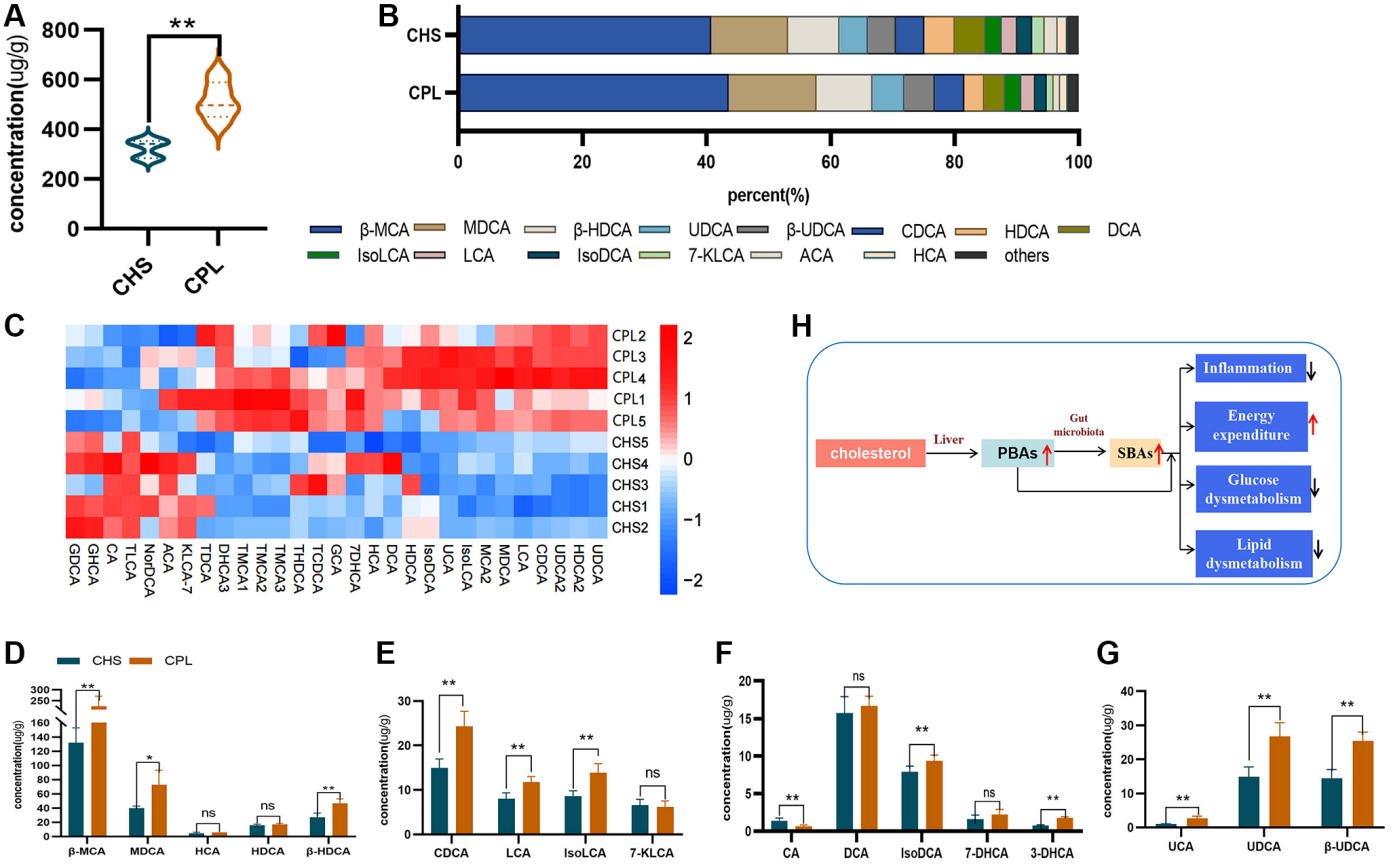
**Effects of CPL on bile acid metabolism in the colonic contents.** (**A**) Total bile acid; (**B**) composition of bile acids. (**C**) Heatmap of bile acids. (**D**) β-MCA and SBAs converted from β-MCA. (**E**) CDCA and SBAs converted from CDCA. (**F**) CA and SBAs converted from CA. (**G**) UCA and SBAs converted from UCA. (**H**) CPL affected gut bile acid metabolism. *n* = 5 mice per group. The data are presented as the mean ± SEM. Statistical analysis was performed using Student’s *t* test. ^*^p < 0.05, ^**^p < 0.01. Abbreviations: ns: not significant; β-MCA: Beta-muricholic acid; MDCA: murideoxycholic acid; HCA: hyocholic acid; HDCA: hyodeoxycholic acid; CDCA: chenodeoxycholic acid; LCA: lithocholic acid; IsoLCA: isolithocholic acid; 7-KLCA: 7-ketolithocholic acid; CA: cholic acid; DCA: deoxycholic acid; IsoDCA: isodeoxycholic acid; 7-DHCA: 7-dehydrocholic acid; 3-DHCA: 3-dehydrocholic acid; UCA: ursocholic acid; UDCA: ursodeoxycholic acid; β-UDCA: beta-ursodeoxycholic acid.

PBAs are produced in the liver with cholesterol and are transformed into SBAs through the intestinal flora [24]. Bile acids are not only vital for glucose and lipid metabolism and are also important signaling molecules for inflammation and energy expenditure (Figure 5H) [24, 25]. In this study, 29 bile acids were detected, and total bile acid levels were greatly increased by CPL treatment (Figure 5A). Only 7 bile acids were decreased, but the levels of 22 bile acids were increased in the CPL group (Figure 5B, 5C).

The top 10 concentrations of bile acid were β-MCA, MDCA, UDCA, β-UDCA, CDCA, HDCA, DCA, isoLCA, LCA and isoDCA in both groups, and the levels of all of these bile acids were increased by CPL treatment (Figure 5B–5G). Four PBAs (including β-MCA, CDCA, CA and UCA) and 13 SBAs (converted from these 4 PBAs) were selected for further study (Figure 5D–5G). The levels of β-MCA, the most important primary bile acid in mice, were significantly increased by CPL treatment, and the levels of SBAs (converted by β-MCA, including MDCA, HCA, HDCA and β-HDCA) were also increased (Figure 5D). The levels of LCA and iso LCA, which are SBAs converted by CDCA, were increased by CPL treatment (Figure 5E). The same changes were observed for UCA, and CPL treatment increased the concentrations of UCA, UDCA and β-UDCA (Figure 5G). As an important primary bile acid in humans, CA, which is present in a low proportion of mouse colon contents, was decreased by CPL treatment (Figure 5F). However, the levels of secondary bile acids (converted by CA, including DCA, isoDCA, 7-DHCA and 3-DHCA), especially isoDCA and 3-DHCA, were increased in the CPL group (Figure 5F).

### CPL affect gut TMA/TMAO metabolism in high-sucrose-induced obese mice

**Figure 6:**
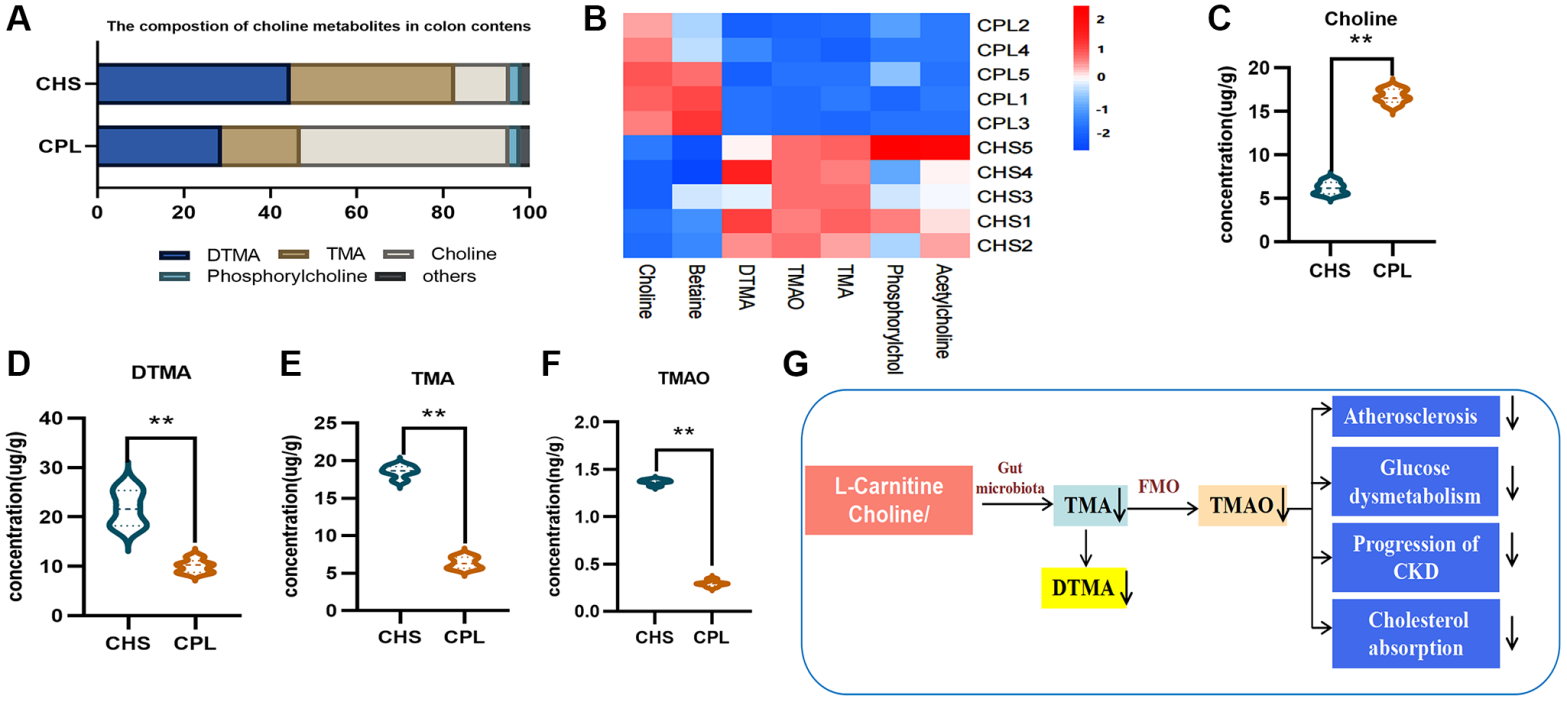
**Effects of CPL on TMA/TMAO metabolism in colonic contents.** (**A**) Composition of choline metabolites. (**B**) Heatmap of choline metabolites. (**C**) Concentration of choline. (**D**) Concentration of DTMA. (**E**) Concentration of TMA. (**F**) Concentration of TMAO. (**G**) CPL affected TMA/TMAO metabolism. *n* = 5 mice per group. The data are presented as the mean ± SEM. Statistical analysis was performed using Student’s *t* test. ^*^p < 0.05, ^**^p < 0.01. Abbreviations: DTMA: dimer trimethylamine; TMA: trimethylamine; TMAO: trimethylamine N-oxide.

In this study, we found that choline remained more abundant in the colonic contents of mice in the CPL group (Figure 6A–6C), which indicated that less choline might be utilized by mice in the CPL group than by those in the CHS group. Correspondingly, the concentration of TMA, a metabolite of choline, was greatly reduced in the CPL group, and the dimer level was also reduced (Figure 6D–6E). TMA enters the blood and is converted to TMAO in the liver. A small amount of TMA can be converted to TMAO by gut microbes. Therefore, TMAO was detected at a low concentration in the colonic contents of mice, and at a lower concentration, it was detected in the CPL group (Figure 6F). Emerging experimental and clinical evidence shows that TMAO may be involved in the etiology of hypertension, atherosclerosis, coronary artery disease, diabetes, and renal failure [26]. Our study showed that CPL might reduce the utilization of choline and decrease the production of TMA, which could benefit the health of obese hosts (Figure 6G).

### FMT experiments validated that CPLs alleviate high-sucrose diet-induced obesity by regulating gut microbes

**Figure 7:**
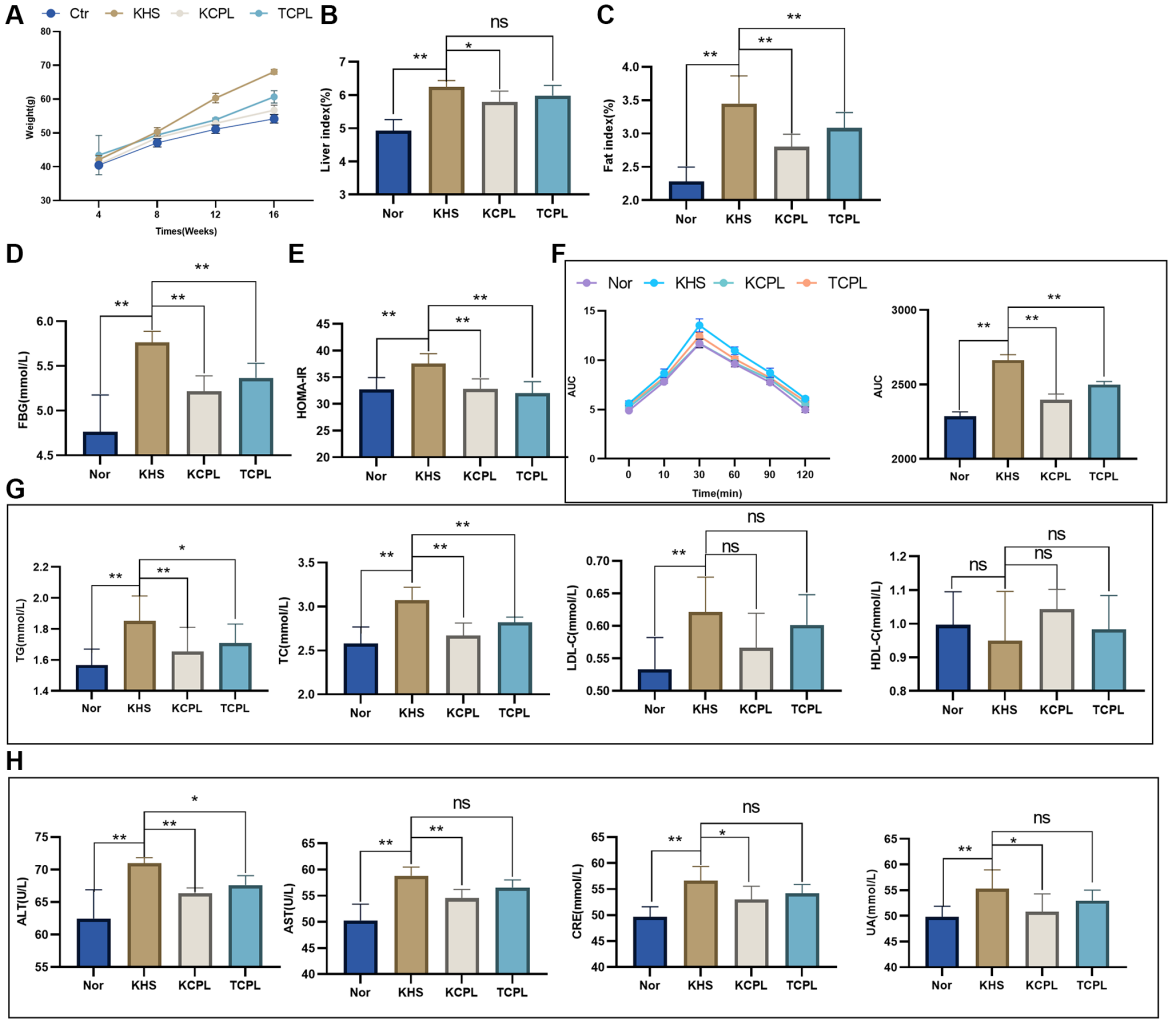
**Fecal microbiota transplantation (FMT) experiments confirmed that CPLs alleviated high-sucrose diet-induced obesity by regulating gut microbes.** (**A**) Weights of the mice. (**B**) Liver indices. (**C**) Fat index. (**D**) Fasting blood glucose (FBG) level. (**E**) Homeostasis model assessment of insulin resistance (HOMA-IR). (**F**) The oral glucose tolerance test (OGTT) and area under the curve (AUC) were calculated for blood glucose levels during the OGTT. (**G**) Serum lipid levels. (**H**) Hepatic and renal function. *n* = 6 mice per group. The data are presented as the mean ± SEM. Statistical analysis was performed using Student’s *t* test. ^*^p < 0.05, ^**^p < 0.01, Abbreviation: ns: not significant.

KM was used to recertify our study. Furthermore, feces from mice in the CPL group were transplanted into mice in the TCPL group. Not surprisingly, CPLs reduced body weight not only in C57BL/6J mice but also in KM mice (Figure 7A, Table 1). Similarly, the liver indices and fat indices were reduced in the CPLs (Figure 7B, 7C). Biochemical indices, including FBG, HOMA-IR and OGTT results, which indicate blood glucose levels, were decreased in the CPLs (Figure 7D–7F). Like in the C57 experiment, TG and TC were significantly decreased by CPLs, but LDL-C and HDL-C did not significantly change (Figure 7G). We also found that CPLs improved hepatic and renal function in KM mice (Figure 7H).

**Table 1:** Dynamic weight in different groups at KM mice confirmatory experiment.

	**0w**	**4w**	**8w**	**12w**	**16w**
Nor	34.1 ± 1.1	40.5 ± 1.1^*^	47.1 ± 1.2^**^	51.1 ± 1.2^**^	54.2 ± 1.3^**^
KHS	34.6 ± 2.1	42.1 ± 1.2	50.4 ± 1.2	60.1 ± 1.4	68.1 ± 0.7
KCPL	35.4 ± 2.3	40.7 ± 0.4	48.6 ± 0.6^**^	52.8 ± 0.6^**^	56.6 ± 1.3^**^
TCPL	34.8 ± 1.1	40.4 ± 2.0	49.4 ± 0.4	54.1 ±0.5^**^	60.7 ± 1.8^**^

Values are means ± SE, *N* = 6 per group. Statistical analysis was performed using one-way ANOVA. ^*^P < 0.05, ^**^P < 0.01 vs. KHS.

More importantly, we observed that mice in the TCPL group (FMT mice) had a lower weight, liver index and fat index. Additionally, the changes in blood glucose and lipid levels and hepatic and renal functions were greater in the TCPL group than in the KHS group (Figure 7), which indicated that CPLs might regulate gut microbes to alleviate high-sucrose diet-induced obesity.

### CPLs affect the metabolism of gut metabolites and the expression of tight junction proteins

**Figure 8:**
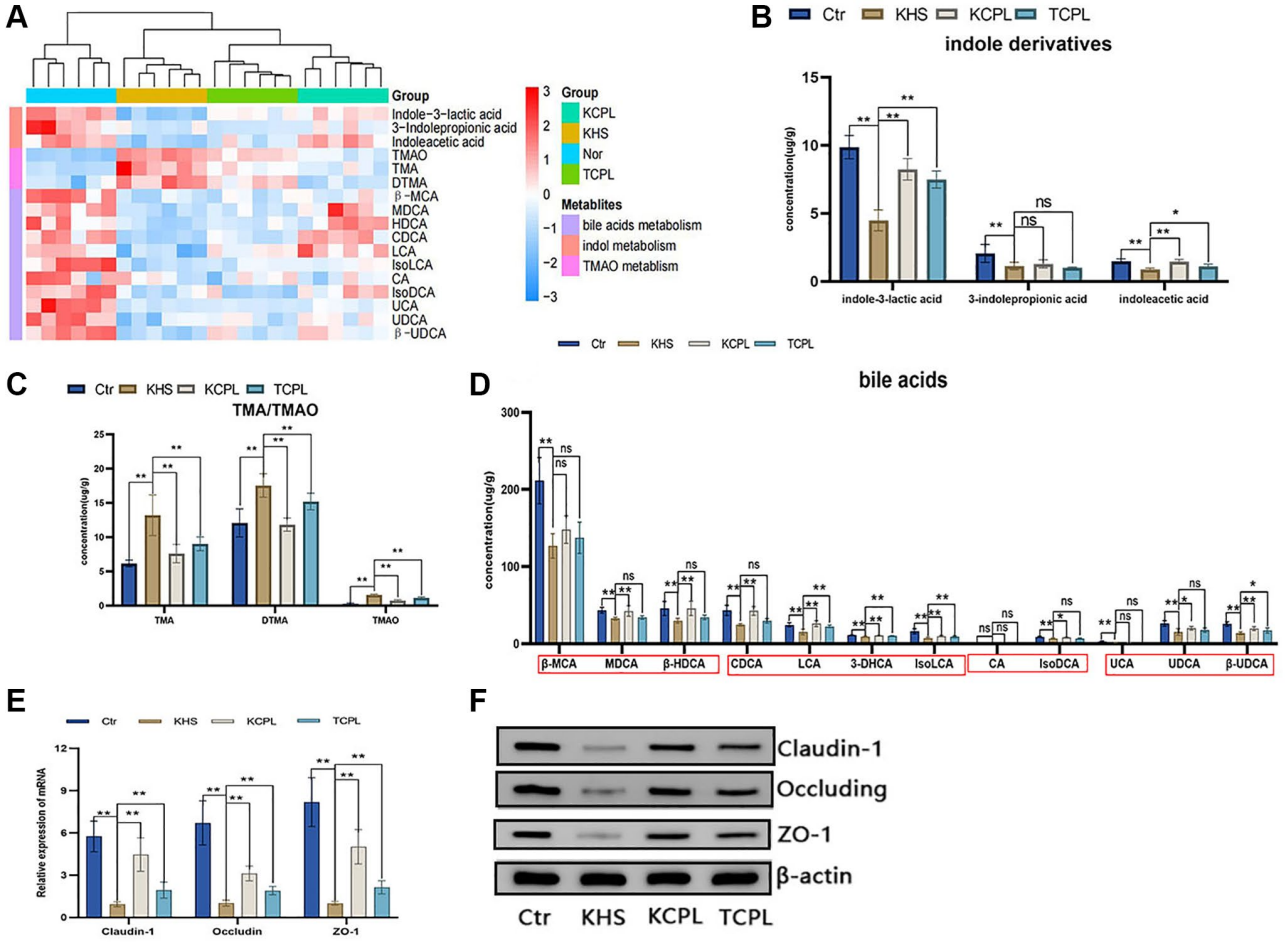
**The levels of eighteen intestinal metabolites and the expression of tight junction proteins were confirmed in the KM experiment.** (**A**) Heatmap of eighteen reconfirmed intestinal metabolites. (**B**) Concentration of indole derivatives. (**C**) Concentration of TMA and TMAO. (**D**) Concentrations of bile acids. (**E**) Relative gene expression of claudin-1, occludin and ZO-1. (**F**) Protein expression of claudin-1, occludin and ZO-1. *n* = 6 mice per group. The data are presented as the mean ± SEM. Statistical analysis was performed using Student’s *t* test. ^*^p < 0.05, ^**^p < 0.01, Abbreviation: ns: not significant.

Eighteen metabolites whose expression significantly changed by high-sucrose diets were detected via redetection in the KM experiment (Figure 8A). Compared to those in the KHS group, the levels of three indole derivatives, indole-3-lactic acid, indoleacetic acid and 3-indolepropionic acid, were greater, although there was no significant difference in the level of indoleacetic acid not only in the TCPL group but also in the KCP group (Figure 8B). The concentrations of TMA and TMAO were strongly decreased in the KCPL and TCPL groups in this confirmatory experiment (Figure 8C). The changes in bile acid levels were similar to the results of the C57 mouse experiment (Figure 8D). In this study, we reconfirmed that CPLs can increase indole derivatives and bile acid levels (especially SBA levels) and decrease TMA and TMAO levels (Figure 8A–8D).

Claudin-1, occludin and ZO-1 are the most important tight junction proteins in the intestinal mucosa. In our study, CPLs upregulated the expression of claudin-1, occludin and ZO-1, which indicated that CPLs could repair the integrity of the intestinal mucosa (Figure 8E, 8F).

## DISCUSSION

Many studies have shown that CPL can reduce fat accumulation and regulate glucolipid metabolism [10]. However, studies on the effects of CPL on sucrose-induced obesity are lacking. This study showed that CPL can ameliorate high-sucrose diet-induced obesity and ameliorate disorders of glucolipid metabolism. What is the underlying mechanism? Our previous study revealed that CCPPs could alleviate T2DM by increasing SCFA-producing bacteria, promoting the production of SCFAs and upregulating SCFA-GLP1/PYY-associated sensory mediators [15]. However, the safety of CCPPs is still unclear, and mechanistic studies on the use of CCPPs for the treatment of T2DM are limited. CPL can be used as teas to induce weight loss and reduce blood glucose levels, and they have been widely used in China for thousands of years [27]. In addition to polysaccharides, compounds isolated and identified from CPL, such as triterpenoids, flavonoids and phenols, have also been shown to be useful for treating metabolic diseases [10, 11, 28]. Hence, we used CPL to prevent high-sucrose diet-induced obesity, which will be a meaningful area of study.

Diets affect the composition of the gut microbiota, which is widely accepted [29]. Excessive intake of sugars can cause many health problems, such as T2DM, obesity, and nonalcoholic fatty liver disease, by altering microbial ecology [19]. In our study, we found that a long-term high-sucrose diet altered nutrient intake, as indicated by decreased protein and lipid intake but increased carbohydrate intake. An unhealthy diet clearly disrupted the gut microbiota composition. However, this gut microbiota disorder could be improved by CPL treatment.

*Firmicutes* and *Bacteroidetes* are the predominant bacterial phyla colonizing the healthy human gut [30]. A decrease in the *Firmicutes*/*Bacteroidetes* ratio in individuals with obesity has been observed in many clinical studies and has become a specific microbial signature of obesity [31]. CPL treatment increased the ratio of *Firmicutes* to *Bacteroidetes* in high-sucrose diet-induced obese mice, which might be beneficial for the health of the host.

Furthermore, we analyzed the changes in the gut microbiota at the family level. *Akkermansiaceae*, a well-known gut microbial family, is beneficial for human health, and the lack or decreased abundance of this commensal bacterium has been linked to multiple diseases [32, 33]. *Akkermansia muciniphila* (a species of *Akkermansiaceae*) is a potential probiotic for the treatment of metabolic disease that has been explored in many studies [32–34]. This study is the first to report that long-term intake of high amounts of sucrose can reduce the abundance of *Akkermansiaceae* and that CPL could increase the abundance of *Akkermansiaceae*. *Lachnospiraceae*, which comprises 58 genera and several unclassified strains, is the main family that has been detected in the human intestine [35]. *Lachnospiraceae*, can produce SCFAs, SBAs and indole derivatives with amnio acids, saccharides and long fatty acids [35]. Our study showed that the abundance of *Lachnospiraceae* was increased by CPL, correspondingly, gut metabolites, including SCFAs, SBAs and indoles, were increased by CPL. Some studies have shown that the abundance of *Prevotellaceae* is negatively related to the progression of chronic kidney disease, Parkinson's disease, depression, and other conditions [36–38]. *Prevotellaceae* can produce butyrate and ameliorate intestinal barrier dysfunction [39]. The abundances of *Prevotellaceae* were decreased by a high-sucrose diet, which might lead to a reduction in the production of beneficial metabolites in the host. CPL can ameliorate gut microbiome disorders. *Muribaculaceae*, also known as family S24-7, are dominant in the mouse gut microbiota but have not been cultured until recently. Hence, the function of *Muribaculaceae* in relation to the host is unclear [40]. Our study showed that a high-sucrose diet increased *Muribaculaceae* abundance, indicating that *Muribaculaceae* might be related to the progression of disease.

In a confirmatory experiment with KM mice, we reconfirmed the effects of reducing weight and improving metabolic disorders through the use of CPL. FMT experiments proved that the effects of CPL on high-sucrose diet-induced obese mice were closely related to gut microbes. Eighteen metabolites, which exhibited a significant change by high sucrose diets, were detected in the confirmatory experiment, and the findings reconfirmed that the addition of CPL increased indole derivatives and bile acids (especially SBAs) and decreased TMA and TMAO. Furthermore, we also found that CPL could upregulate the expression of claudin-1, occludin and ZO-1 and restore the integrity of the intestinal mucosa.

Humans have evolved intimate symbiotic relationships with a consortium of gut microbes, and these microbes use nutrients in the host intestinal tract to subsist [41]. Gut microbe gene functional prediction revealed that CPL could improve the metabolic capacity of gut microbes to produce more gut metabolites. Gut metabolites, which are produced by gut microbes with gut nutrients, play important roles in host health. In this study, amnio acids and analogs, fatty acids and metabolites, carbohydrates and metabolites, tryptophan-related metabolites, TMA/TMAO-related metabolites, and bile acids were absolutely quantified. CPL treatment reversed high-sucrose diet-induced gut microbiota disorders, and these changes were accompanied by alterations in gut metabolites.

Indole-3-lactic acid, indoleacetic acid and 3-indolepropionic acid are the main tryptophan metabolites found in the colon, and their levels significantly increase after CPL treatment. Indoles and indole derivatives act as aryl hydrocarbon receptors to repair the epithelial barrier and maintain intestinal immunostability. These compounds are implicated in disease etiology and affect the progression of cancers, T2DM, Alzheimer’s disease and other diseases [42, 43]. TMA is a molecule generated from choline, betaine, and carnitine via gut microbial metabolism and is rapidly further oxidized by hepatic flavin monooxygenases to form TMAO [44]. Recent studies have shown a positive correlation between elevated plasma levels of TMAO and an increased risk of CVD, T2DM and chronic kidney disease [44, 45]. CPL decreased the utilization of choline, as indicated by decreased TMA/TMAO production.

Recently, an increasing number of researchers have focused on the effects of bile acids on pathophysiological processes [46, 47]. In this study, PBAs, including β-MCA, CDCA and UCA, and SBAs, including LCA, iso-LCA, iso-DCA, 3-DHCA, UDCA and β-UDCA, were significantly increased by CPL. Bile acids are complex and important signaling molecules for the host, but their ability to regulate glucolipid metabolism is accepted by the public. LCA and iso-LCA are effective ligands for farnesoid X receptor and G protein-coupled receptor 5 and have beneficial effects on diabetes. UDCA is a well-known drug for the treatment of cholestatic liver disease, but it is also considered a promising therapeutic approach for inflammatory bowel diseases, hypertransaminasemia and colitis-associated cancer [47, 48].

**Figure 9:**
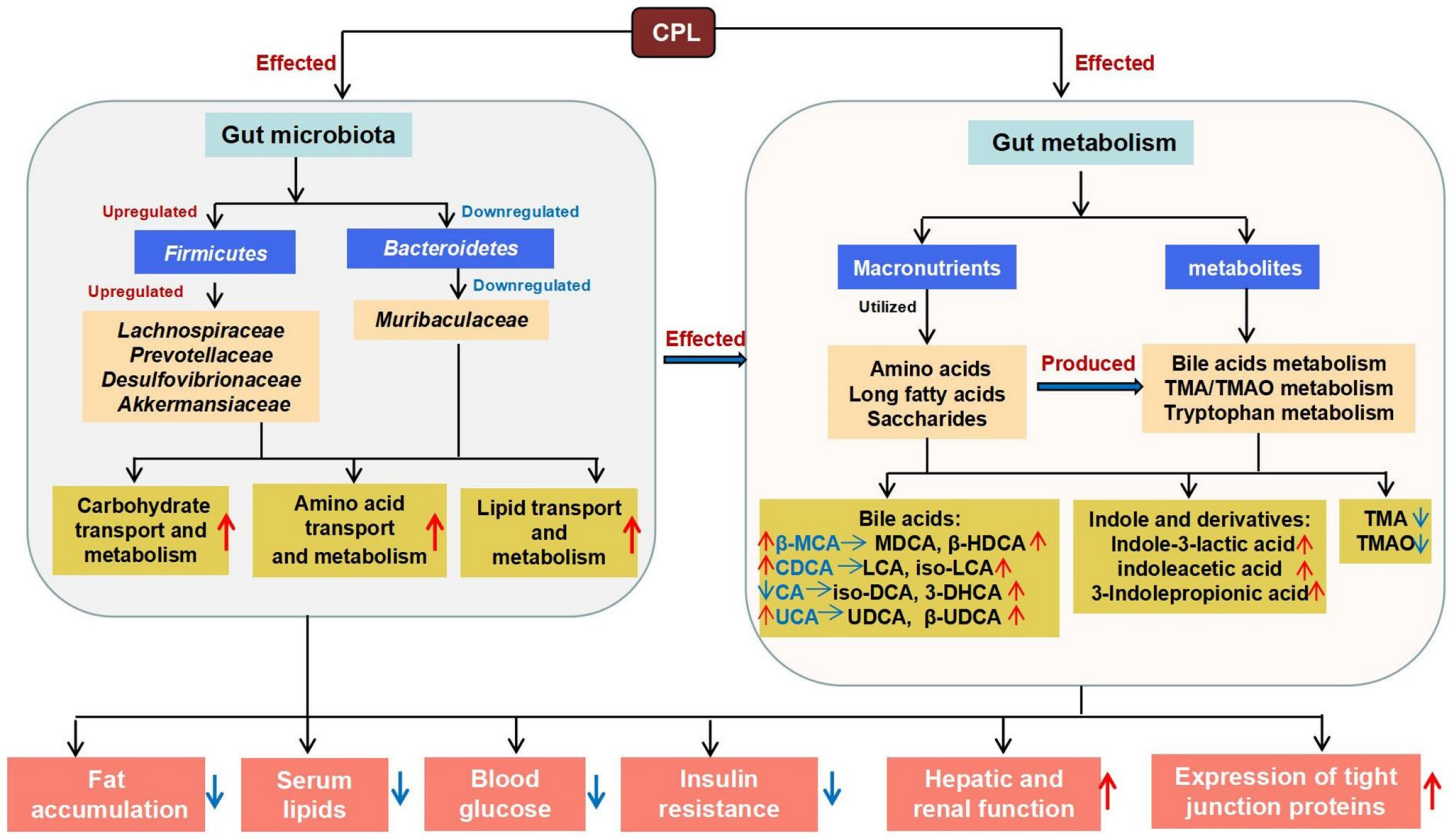
The mechanism by which CLPs improve high-sucrose diet-induced obesity.

In summary, this study revealed that long-term high-sucrose intake markedly disrupts the gut microenvironment and suggested that controlling sucrose intake might be vital to human health. Fortunately, CPL have the ability to prevent sucrose-induced obesity and ameliorate disorders of the gut microbiota and metabolites (Figure 9). Thus, CPL can be considered an important supplement for improving sweet food-caused metabolic diseases.

## Supplementary Materials

Supplementary Figure 1

Supplementary Tables

Supplementary File 1

Supplementary File 2

Supplementary File 3
