# Supplementary material for: Cyclocarya paliurus leaves alleviate high-sucrose diet-induced obesity by improving intestinal metabolic disorders
Source: Aging (Albany NY). 2024 Mar 14;16(6):5452–70. doi: 10.18632/aging.205657 (PMC11006468; doi:10.18632/aging.205657)
Supplement: Supplementary Figure 1 [file aging-16-205657-s001.pdf]

SUPPLEMENTARY FIGURE

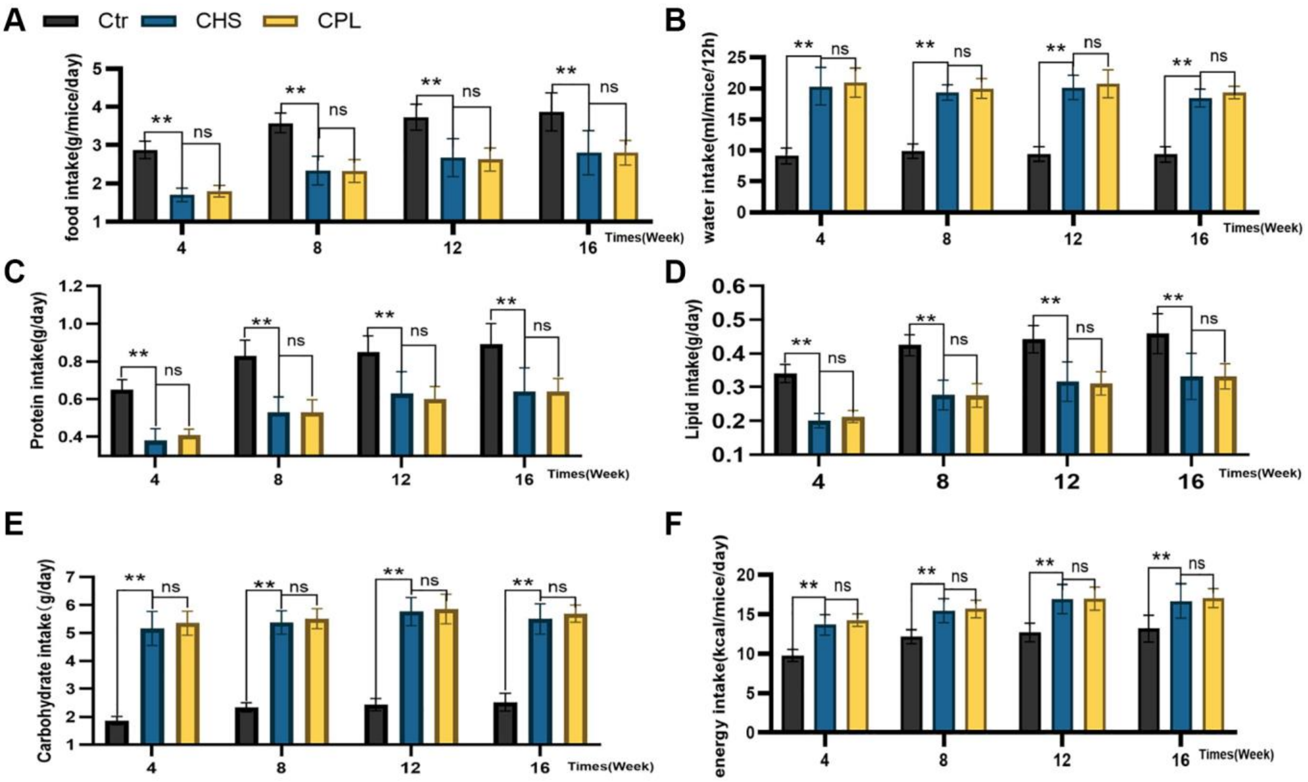

Supplementary Figure 1. A high-sucrose diet influences energy intake. (**A**) Food intake. (**B**) Water intake. (**C**) Protein intake. (**D**) Lipid intake. (**E**) Carbohydrate intake. (**F**) Energy intake. n = 10 mice per group. The data are presented as the mean ± SEM. Statistical analysis was performed using Student's t test. $^{*}p < 0.05$, $^{**}p < 0.01$, Abbreviation: ns: not significant.
