# Supplementary material for: Cyclocarya paliurus leaves alleviate high-sucrose diet-induced obesity by improving intestinal metabolic disorders
Source: Aging (Albany NY). 2024 Mar 14;16(6):5452–70. doi: 10.18632/aging.205657 (PMC11006468; doi:10.18632/aging.205657)
Supplement: Supplementary Tables [file aging-16-205657-s002.pdf]

SUPPLEMENTARY TABLES

Supplementary Table 1. Nutritional ingredients of normal diet.

Vitamin		Amino acid		Mineral element		Ratio of energy	
Vitamin A, IU	7800.00	Met+Cys,g	5.80	Na, g	3.10	Protein	23.07%
Vitamin D, IU	1200.00	Lys, g	8.90	Mg, g	2.90	Lipid	11.85%
Vitamin E, mg	87.00	Trp, g	2.10	K, g	7.40	Carbohydrate	65.08%
Vitamin K, mg	5.00	Arg, g	9.90	Cu, mg	11.40	Total energy	3.40 kcal/g
Vitamin B1, mg	10.00	Leu, g	14.80	Fe, mg	113.70		
Vitamin B2, mg	15.00	Ile, g	7.40	Mn, mg	80.00		
Vitamin B6, mg	10.00	Thr, g	6.60	Zn, mg	31.60		
Vitamin B12, mg	0.02	Val, g	8.90	Se, mg	0.20		
Niacin, mg	55.00	His, g	4.90	I, mg	0.70		
Pantothenic acid, mg	22.00	Phe+Tyr, g	14.60				
Biotin, mg	0.20						
Choline, mg	1250.00						
Folic acid, mg	6.60						

Raw material components: corn, soybean, fish power, flour, yeast power, oil, vitamin and mineral element. Nutritional ingredients(/kg). Above data were offered by Beijing Ke-ao-xie-li Feed Co., Ltd.

Supplementary Table 2. Specific primers for the qRT-PCR analysis.

Gene	Forward	Reverse
Claudin-1	F: GCTGGGTTTCATCCTGGCTTCTC	CCTGAGCGGTCACGATGTTGTC
Occludin	TGGCTATGGAGGCGGCTATGG	ACTAAGGAAGCGATGAAGCAGAAGG
ZO-1	AACCCGAAACTGATGCTGTGGATAG	CGCCCTTGGAATGTATGTGGAGAG
