# Supplementary material for: Cyclocarya paliurus leaves alleviate high-sucrose diet-induced obesity by improving intestinal metabolic disorders
Source: Aging (Albany NY). 2024 Mar 14;16(6):5452–70. doi: 10.18632/aging.205657 (PMC11006468; doi:10.18632/aging.205657)
Supplement: Supplementary File 3 [file aging-16-205657-s005.pdf]

Supplementary File 3. Supplementary methods.

16S rRNA gene sequencing analysis

Colonic content samples were collected for 16S rRNA gene analysis. Briefly, the steps of the analysis were DNA extraction, DNA quality detection, 16S rRNA gene amplification, purification of the PCR products, PE library construction, Illumina sequencing and bioinformatics analysis[20, 21].

A TIANmap Stool DNA Kit (DP328, TIAGEN Biotech Co., Ltd., Beijing, China) was used to extract genomic DNA from the colon contents according to the manufacturer's protocols. The concentration and purity of the extracted DNA were determined with a TBS-380 Mini-Fluorometer (Turner BioSystems Co., Ltd., USA) and a NanoDrop2000 (Thermo Scientific Co., Ltd., USA), respectively. The DNA extraction quality was confirmed on a 1% agarose gel. The V3-V4 regions of the bacterial 16S rRNA gene were amplified with the following sets of primers: 338F, 5-ACTCCTACGGGAGGCAGCAG-3; and 806R, 5'-GGACTACHVGGGTWTCTAAT-3' (ABI GeneAmp® 9700, USA). The PE library was constructed after purification, quantification and normalization of the PCR products. Finally, the eligible library was sequenced via the Illumina MiSeq platform. The paired-end (PE) reads, which were obtained from the Illumina MiSeq platform, were used for further bioinformatics analysis. PE read splicing, tag filtering and removal of chimeras were performed before bioinformatics analysis. The optimized reads were put into Uparse (version v. 7.0.1090, http://www.drive5.com/uparse/) software to obtain operational taxonomic units (OTUs) with a 97% similarity threshold, and the OTUs were taxonomically annotated based on Silva 138 (https://www.arb-silva.de/) at the domain, kingdom, phylum, class, order, family, genus, and species levels. The alpha diversity (including the Shannon index, ACE index and Chao1 index) was estimated by Mothur (version v. 1.30.2, https://www.mothur.org/wiki/Download_mothur) software. Beta diversity analysis, including principal component analysis (PCA) and hierarchical clustering, was performed using the Bray–Curtis algorithm of QIIME and R software to compare the similarity of species diversity in different groups. In addition, COG functional prediction by PICRUSt (http://picrust.github.io/picrust) was used to analyze the metagenomic function of the gut microbiota. These data were analyzed by Shanghai Majorbio BioPharm Technology Co., Ltd.

Quantitative analysis of intestinal metabolites

The colonic contents were collected for quantitative analysis via UHPLC–QTOF–MS technology. In the C57 experiment, 650 metabolites were detected in this study. In the KM experiment, 3 indole derivatives (including indole-3-lactic acid, indoleacetic acid and 3-indolepropionic acid), 12 bile acids (including β-MCA, MDCA, β-HDCA, CDCA, LCA, IsoLCA, CA , IsoDCA, UCA, UDCA, β-UDCA, 3-DHCA) and TMA related metabolites (including DTMA, TMA and TMAO) were absolutely quantified with UHPLC–QTOF–MS technology. The steps included sample preprocessing, QC preparation, standard curve preparation, LC–MS/MS mass spectrometry analysis and data processing. These steps were performed by Shanghai Applied Protein Technology Co., Ltd.. A total of 100 mg of colon contents was added to precooled methanol+acetonitrile+water solution (2:2:1, v/v), vortexed for 30 sec, ultrasonicated at 4°C for 30 min, incubated at -20°C for 10 min, and centrifuged at 14000 × g for 20 min. The supernatant was vacuum dried for further analysis. The dried supernatant was dissolved in an acetonitrile solution (acetonitrile: water =1:1, v/v) for LC–MS/MS analysis.

The 650 gut metabolites were weighed accurately, and 8 different concentrations of each metabolite were prepared with acetonitrile. After many cycles, the best chromatography and mass spectrometry conditions were used. We used an Agilent 1290 Infinity LC ultrahigh-performance liquid chromatography system (UHPLC) with HILIC and a C18 column for separation. The HILIC column chromatography conditions used were as follows: column temperature, 35°C; flow rate, 0.3 mL/min; mobile phase A: 90% water + 2 mM ammonium formate +10% acetonitrile; mobile phase B: methanol + 0.4 formic acid. The gradient elution procedure was as follows: 0-1.0 min 85% B, 1.0-3.0 min B was linearly reduced to 80%, 3.0-4.0 B was linearly reduced to 70%, 6.0-10.0 min B was linearly reduced to 50%, 10.0-15.5 min B was linearly reduced to 50%, 15.5-15.5 min B was linearly increased to 85%, and 15.6-23.0 min B was linearly increased to 85%. The C18 column chromatography conditions used were as follows: column temperature, 40°C; flow rate, 0.34 mL/min; mobile phase A: water + 2 mM ammonium acetate + 0.2% ammonium hydroxide; and mobile phase B: 99.5% acetonitrile + 0.5% ammonium hydroxide. The gradient elution procedure was as follows: 0-5.0 min B was linearly increased from 5% to 60%, 5.0-11.0 B was increased to 100%, 11.0-13.0 B 100%, and 13.0-13.1 B was linearly decreased from 100% to 5%. 13.1-16.0 min B 5%. An Agilent 6500+QTRAP mass spectrometer was used for mass spectrum analysis. The conditions were as follows: source temperature: 580°C, ion source gas1 (GS1): 45, ion Source Gas2 (GS2): 60, curtain gas (CUR): 35, IonSpray Voltage (IS): +4500 V or -4500 V in positive or negative modes. Then, based on the peak area and different concentrations of standards, we drew standard curves of 650 gut metabolites, and all the standard curves were saved in File S.1. The colon contents were analyzed via the same chromatography and mass spectrometry methods. Finally, based on the peak areas and standard curves, we calculated the concentrations of each gut metabolite.
